# No advantage of single day 6 good-quality blastocyst transfer versus single day 5 poor-quality blastocyst transfer in frozen-thawed cycles stratified by age: a retrospective study

**DOI:** 10.1186/s12884-023-05387-x

**Published:** 2023-01-30

**Authors:** Yuxia He, Yan Tang, Haiying Liu, Jianqiao Liu, Yuling Mao

**Affiliations:** 1 Department of Obstetrics and Gynecology, Center of Reproductive Medicine, Guangdong Provincial Key Laboratory of Major Obstetric Diseases, The Third Affiliated Hospital of Guangzhou Medical University, 63 Duobao Road, Guangzhou, Guangdong China; 2 Key Laboratory for Reproductive Medicine of Guangdong Province, The Third Affiliated Hospital of Guangzhou Medical University, Guangzhou, Guangdong China; 3 Department of Obstetrics and Gynecology, Center of Reproductive Medicine, Zhongshan City People’s Hospital, Zhongshan, China

**Keywords:** Good-quality blastocyst, Poor-quality blastocyst, Transfer strategy, Live birth rate, Neonatal outcomes

## Abstract

**Background:**

Blastocyst developmental speed, morphological grading and patient age are associated with pregnancy outcomes of frozen-thawed cycles. This study aimed to compare the clinical and neonatal outcomes between poor-quality D5 blastocysts and good-quality D6 blastocysts stratified by patient age.

**Methods:**

A total of 1,623 cycles were divided into two groups: group A (*n* = 723) received one D5 poor-quality blastocyst; group B (*n* = 900) received one D6 good-quality blastocyst. Pregnancy and neonatal outcomes were compared among the four groups stratified by 35 years of age.

**Results:**

When patients were in the same age group, there was no significant difference in terms of age, body mass index, infertility duration, infertility type, fertilization method, proportion of endometrial preparation protocols, and endometrial thickness between D5 poor-quality and D6 high-quality blastocysts groups. Live birth rate of D5 poor-quality blastocysts was higher than that of D6 high-quality blastocysts for patients aged < 35 years (35.48% vs. 31.13%, *p* > 0.05), but there was no statistical difference. The same trend was showed for patients aged ≥ 35 years (29.09% vs. 21.28%, *p* > 0.05). Moreover, when patients were in the same age category, there was no significant difference in terms of gestational age, birth weight, birth height, and rates of preterm birth, low birth weight, and very low birth weight between groups A and B.

**Conclusions:**

The preferential selection of poor-quality D5 blastocysts for transfer compared to high-quality D6 blastocysts is recommended, especially for advanced age patients. Single good-quality D6 blastocyst transfer can be considered for the acceptable live birth rate.

## Background

Multiple embryo transfer has been preferentially performed to increase pregnancy outcomes for a few decades after the first baby was born using in vitro fertilization and embryo transfer technology (IVF-ET) [1]. However, this approach has resulted in a significantly increased risk of multiple pregnancies. Multiple pregnancy is one of the most common complications of IVF and is associated with increased maternal complications and neonatal morbidity, as well as financial burden [2, 3]. Therefore, the ultimate focus of IVF has shifted from pursuing pregnancy rate to delivering a single healthy child. Single embryo transfer is the most direct and effective approach for minimizing multiple pregnancies. Currently, single blastocyst transfer (SBT) is preferred in clinical practice because clinical pregnancy and live birth rates are significantly higher than those of single cleavage-stage embryo transfer [4, 5]. However, not all populations are suitable for SBT, as there are many factors that affect pregnancy outcomes that need to be comprehensively considered before deciding how many embryos to transfer [6]. Currently, when patients have blastocysts with different developmental speed and embryo quality, selecting a blastocyst with excellent implantation potential for transfer is a challenge for clinicians to obtain a satisfactory pregnancy outcome and reduce multiple pregnancies.

Blastocyst developmental speed is regarded as one of the important factors affecting IVF pregnancy outcomes. Many studies have shown that the pregnancy outcomes of day 5 (D5) blastocysts are significantly better than those of day 6 (D6) blastocysts in frozen embryo transfer (FET) cycles [7–9], and some meta-analyses have also found that the clinical pregnancy and live birth rates of D5 blastocysts were superior to those of D6 blastocysts [10, 11]. However, blastocyst morphological grading, another parameter that plays a major role in clinical outcomes, still needs to be considered when selecting optimal blastocysts for transfer. Studies have shown that when the blastocysts developed into the same stage, the pregnancy outcomes of high-quality blastocysts are significantly higher than those of poor-quality blastocysts [12, 13]. Blastocyst developmental speed and morphological grading were associated with pregnancy outcomes of IVF, but no study has explored the transfer strategy for the selection of poor-quality D5 blastocysts versus high-quality D6 blastocysts.

The blastocyst transfer strategy should also consider patient age, as live birth rate gradually decreases with age [14]. In clinical practice, double embryo transfer is usually performed to improve the pregnancy outcomes of IVF for advanced age patients, but this decision is accompanied by an increased risk of multiple pregnancies. Our previous study showed that single D5 blastocyst transfer, even for poor-quality blastocysts, could be recommended for patients aged > 35 years because of the acceptable pregnancy outcomes and significantly reduced multiple birth rate [15]. However, no study has explored the transfer strategy for D6 blastocysts based on morphological grading and patient age. Especially for elderly patients, single D6 good-quality blastocyst transfer could also be recommended while maintaining an acceptable pregnancy rate.

Therefore, this retrospective compared the pregnancy outcomes between poor-quality D5 blastocyst transfer and good-quality D6 blastocysts stratified by 35 years of age, and to determine whether single D6 good-quality blastocyst was adequately recommended for all populations undergoing IVF. We hope to provide additional references for selecting a more appropriate blastocyst transfer strategy that reduces multiple pregnancy rate without compromising the live birth rate.

## Materials and methods

### Study population and grouping

This was a retrospective cohort analysis of patients who underwent FET cycles at the Department of Reproductive Medicine of the Third Affiliated Hospital of Guangzhou Medical University between January 2014 and December 2020. The inclusion criteria were as follows: (i) aged 20–39 years; (ii) first IVF/intracytoplasmic sperm injection cycle, (iii) number of ET cycles ≤ 2, and (iv) endometrial thickness ≥ 7 mm on the initiation of progesterone exposure. The exclusion criteria were as follows: (i) blastocysts derived from vitrified or donated oocytes; (ii) preimplantation genetic testing cycles; (iii) stage III to IV endometriosis/adenomyosis; (iv) known uterine anomalies; and (v) untreated hydrosalpinx/uncontrolled systemic diseases prior to FET. Institutional review board approval was obtained from the ethics committee of the Third Affiliated Hospital of Guangzhou Medical University [Ethic no. (2022)-013]. The patients/participants provided their written informed consent to participate in this study.

The FET cycles that met the inclusion criteria were divided into four groups: group A (*n* = 723) received one D5 poor-quality blastocyst; group B (*n* = 900) received one D6 good-quality blastocyst. A total of 29 patients contributed two SBT cycles during the study period. The clinical and neonatal outcomes were analyzed among the four groups stratified by age using a cutoff of 35 years.

### Controlled ovarian stimulation

The patients in this study were treated with either gonadotropin-releasing hormone (GnRH) agonist or GnRH antagonist for pituitary suppression, as previously described [15]. Briefly, individualized starting dosages of recombinant human follitropin (r-hFSH; GONAL-f®, Merck Serono, Switzerland, or Puregon®, MSD, Netherlands) were given according to patient’s age, body mass index (BMI) and ovarian reserve, and follicular development were monitored using transvaginal ultrasonography and serum hormones determinations. Human chorionic gonadotrophin (HCG; Lizhu Group Co., China, or Merck Serono) and/or GnRHa was triggered to induce oocyte maturation when at least three follicles ≥ 17 mm. Oocyte retrieval was performed 36–38 h after administration of HCG and/or GnRHa, and oocytes were incubated for insemination by conventional IVF or ICSI as determined by sperm quality.

As previously described [16], fertilization was checked about 16 h after insemination/injection and was determined by the presence of two pronuclei (2PN). Embryos were placed into the incubator (K-MINC-1000, Cook, United States) and cultured at 6% CO_2_, 5% O_2_ and 37℃. G-1™ plus (Vitrolife, Sweden) was used for cleavage stage embryos and G-2™ plus (Vitrolife, Sweden) for blastocyst stage. The quality of day 3 cleavage embryo was assessed about 68 h post insemination/injection, and the quality of blastocysts were evaluated on Day 5 about 116 h post insemination/injection or Day 6 about 140 h post insemination/injection.

### Embryo grading, vitrification, and warming

**Fig. 1:**
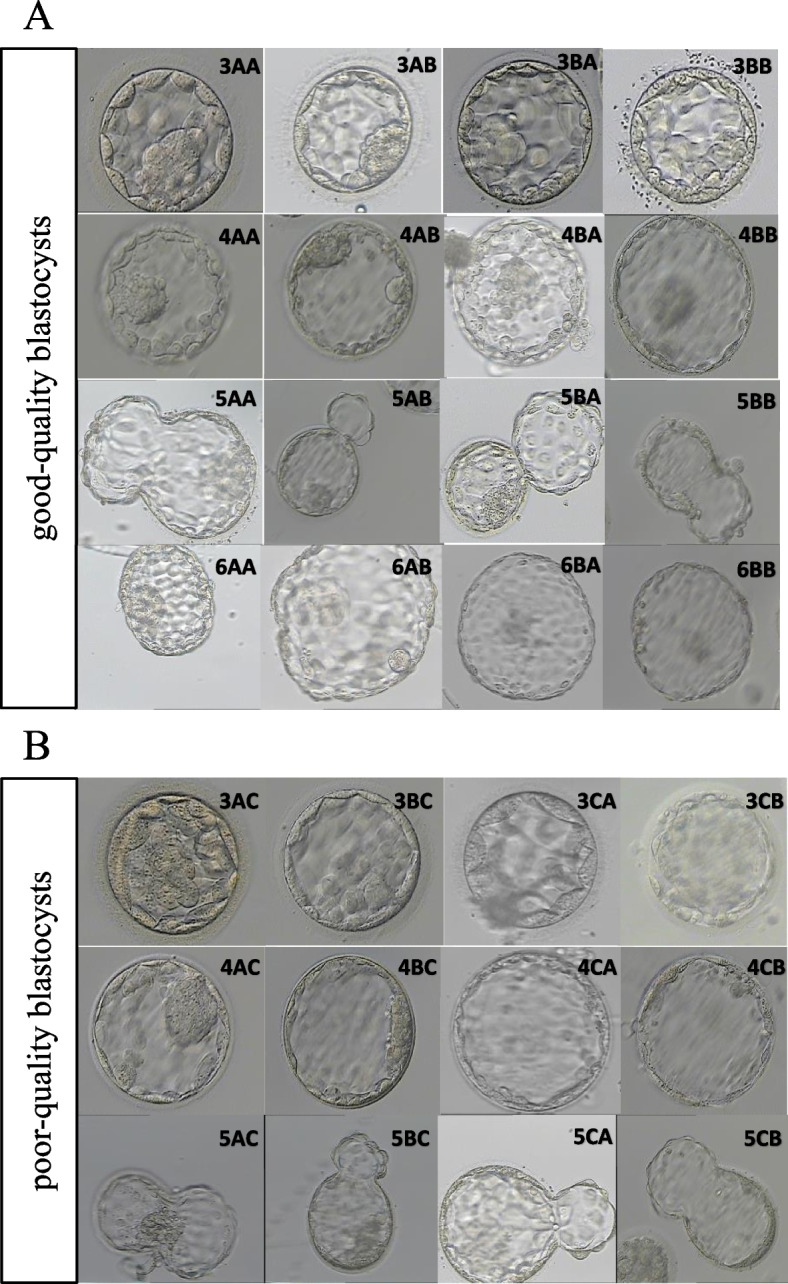
Blastocyst morphological grading. (**A**) Good-quality blastocysts; (**B**) Poor-quality blastocysts, stage 6 non-quality blastocysts were not shown as these blastocysts were not transferred in this study

Blastocyst morphological grading was assessed on day 5 or day 6 using the Gardner and Schoolcraft grading system [17], which depends on blastocyst expansion, inner cell mass development, and trophectoderm appearance. Briefly, blastocyst stage was graded 1 is an embryo in which the blastocoele occupies less than half of the volume; stage 2 is an embryo with the blastocoele occupied half of or greater than half of the volume of the embryo; stage 3 is a full blastocyst with the blastocoele completely filling the embryo; stage 4 is an expanded blastocyst with the blastocoele volume larger than that of the early embryo, with a thinning zona; stage 5 is a hatching blastocyst with the trophectoderm starting to herniate though the zona; and stage 6 is a hatched blastocyst in which the blastocyst has completely escaped from the zona. The inner cell mass was assessed as follows: A, tightly packed, many cells; B, loosely grouped, several cells; or C, very few cells. The trophectoderm was assessed as follows: A, many cells forming a cohesive epithelium; B, few cells forming a loose epithelium; or C, very few large cells. Good-quality blastocysts were graded as AA, AB, BA, and BB with an expansion grade ≥ 3 (Fig. 1A), and the remaining available embryos were regarded as poor-quality blastocysts (Fig. 1B). Blastocysts with a poor morphological score (CC) or low expansion grade (grades 1–2) were not vitrified for subsequent transfer. The precise vitrification and thawing protocol for the blastocysts was performed according to the manufacturer’s instructions (Kitazato Biophama Co. Ltd. Shizuoka. Japan), as previously described [16]. The thawed blastocysts were cultured at 37 °C in a 6% CO2, 5% O2, and 89% N2 incubator for another 4–5 h before transfer.﻿

### Endometrial preparation and embryo transfer

Two main schemes for endometrial preparation in this study were applied according to clinical judgment and patient preference. Natural cycle was performed for patients with regular menstrual cycle. Ovulation was confirmed by transvaginal ultrasonography and serum hormone measurement. On the day of ovulation, the patient received 90 mg vaginal progesterone (Crinone, Merck Serono, Germany) once daily. In artificial cycles, 6 mg oral estrogen (progynova, Bayer, Germany) daily was started on the second to fourth day of the menstrual cycle for one week and adjusted to 8–10 mg based on endometrial thickness and serum hormone levels. Vaginal progesterone (Crinone, Merck Serono, Germany) 90 mg daily and dydrogesterone (Duphaston, Abbott Biologicals B. V., Netherlands)10 mg twice daily were administered for endometrial transformation, as soon as the endometrial thickness reached ≥ 7 mm.

The blastocyst transfer was performed on the sixth day after ovulation confirmation or progesterone exposure using a soft-tipped Wallace (PortexLed., Hythe, United Kingdom) catheter under ultrasound guidance. All the patients received progesterone for luteal support and continued at the same doses until 14 days after FET and up to 10 weeks of gestational age in cases of pregnancy.

### Outcome parameters

Primary outcome was live birth rate, and secondary outcomes included multiple pregnancy rate, clinical pregnancy rate, early miscarriage rate, and neonatal outcomes. Live birth was defined as the delivery of a live fetus after 28 weeks of gestation. Clinical pregnancy was defined as at least one intrauterine or extrauterine gestational sac confirmed by ultrasound at 6–7 weeks of gestation. Early miscarriage was defined as intrauterine pregnancy loss before 12 weeks of gestation. Preterm birth was defined as delivery of a viable infant before 37 weeks of gestation. Low birth weight was defined as a birth weight of less than 2500 g and very low birth weight as a birth weight of less than 1500 g.

### Statistical analysis

All data were analyzed using Statistical Package for Social Science version 22.0 software (IBM, NY, USA). Student’s t-test was used for continuous variables, which were expressed as the mean ± standard deviation. The Fisher’s exact test or chi-square test, as appropriate, was used for categorical variables, which were described as frequencies and percentages. Statistical significance was set at *p* < 0.05.

## Results

A total of 1,623 FET cycles were included in this study from January 2014 to December 2020 and divided into two groups, depending on the blastocyst quality and developmental day. Two pregnant patients were lost to follow-up in this study. Clinical and neonatal outcomes were analyzed among these groups stratified by 35 years of age. The baseline patient characteristics are shown in Table 1. When the patients were in the same age category, there was no significant difference in terms of age, body mass index, infertility duration, infertility type, fertilization method, proportion of endometrial preparation protocols, and endometrial thickness between D5 poor-quality and D6 high-quality blastocysts groups.

**Table 1:** Baseline characteristics of the patients among groups A-B stratified by age

	< 35 years of age			≥ 35 years of age		
	Group A	Group B	*P*	Group A	Group B	*P*
Cycles (n)	558	665		165	235	
female age(year)	29.84 ± 2.75	29.93 ± 2.86	0.274	36.65 ± 1.41	36.65 ± 1.37	0.498
BMI (kg/m2)	21.83 ± 3.31	21.47 ± 3.13	0.154	22.43 ± 3.69	22.20 ± 3.73	0.742
Infertility duration (years)	4.01 ± 2.44	3.98 ± 2.37	0.454	5.52 ± 3.75	5.28 ± 3.72	0.284
Type of infertility			0.652			0.754
Primary infertility	58.42 (326/558)	57.14 (380/665)		32.12 (53/165)	33.62 (79/235)	
Secondary infertility	41.58 (232/558)	42.86 (285/665)		67.88 (112/165)	66.38 (156/235)	
Fertilization method			0.160			0.259
IVF	79.57 (444/558)	75.04 (499/665)		84.85 (140/165)	85.53 (201/235)	
ICSI	16.85 (94/558)	20.15 (134/665)		12.12 (20/165)	13.19 (31/235)	
IVF + ICSI	3.58 (20/558)	4.81 (32/665)		3.03 (5/165)	1.28 (2/235)	
Endometrial preparation (%)			0.061			0.276
Natural	31.72 (177/558)	36.84 (245/665)		48.48 (80/165)	42.98 (101/235)	
HRT	68.28 (381/558)	63.16 (420/665)		51.52 (85/165)	57.02 (134/235)	
Endometrial thickness (mm)	8.96 ± 1.42	9.07 ± 1.46	0.085	8.89 ± 1.44	9.05 ± 1.46	0.956

Group A, one poor-quality day 5 blastocyst

Group B, one good-quality day 6 blastocyst

The clinical outcomes of the patients between the two groups stratified by 35 years of age are presented in Table 2. For women younger than 35 years, there was no significant difference in the rates of clinical pregnancy (42.41% vs. 44.80%), live birth (31.13% vs. 35.48%), and early miscarriage (21.63% vs. 17.60%) between groups B and A. In women aged 35 years and older, patients in group B had a non-significant trend toward a lower live birth rate than group A (21.28% vs. 29.09%). Additionally, the early miscarriage rate was not statistically different between the two groups (33.33% vs. 31.58%). Moreover, subgroup analysis of clinical outcomes within the advanced age patients was performed. As presented in Table 3, for patients aged 35 to 37 years old, patients in group B had a non-significant trend toward a lower live birth rate than group A (21.82% vs. 30.36%). This result is also applicable to patients aged 38 to 39 years old (20.0% vs. 26.42%).

**Table 2:** The clinical outcomes of the patients in groups A-B stratified by 35 years of age

	< 35 years of age			≥ 35 years of age		
	Group A	Group B	*P*	Group A	Group B	*P*
Cycles (n)	558	665		165	235	
Pregnancy rate	44.80 (250/558)	42.41 (282/665)	0.400	46.06 (76/165)	33.19 (78/235)	0.009
Live birth rate	35.48 (198/558)	31.13 (207/665)	0.107	29.09 (48/165)	21.28 (50/235)	0.074
Singleton	35.30 (197/558)	30.83 (205/665)	0.097	29.09 (48/165)	20.85 (49/235)	0.058
Twin	0.18 (1/558)	0.30 (2/665)	1.000	0 (0/165)	0.43 (1/235)	1.000
Monozygotic twins rate	1.60 (4/250)	3.19 (9/282)	0.235	6.58 (5/76)	2.56 (2/78)	0.419
Early abortion rate	17.60 (44/250)	21.63 (61/282)	0.244	31.58 (24/76)	33.33 (26/78)	0.816
Abortion rate	19.60 (49/250)	24.82 (70/282)	0.149	35.53 (27/76)	35.90 (28/78)	0.962
Ectopic pregnancy rate	1.20 (3/250)	1.06 (3/282)	1.000	1.32 (1/76)	0 (0/78)	0.494

*P* < 0.05 was considered statistically significant

**Table 3:** Subgroup analysis of clinical outcomes within the advanced age patients

	35–37 years old			38–39 years old		
	Group A	Group B	*P*	Group A	Group B	*P*
Cycles (n)	112	165		53	70	
Pregnancy rate	45.54 (51/112)	32.12 (53/165)	0.024	47.17 (25/53)	35.71 (25/70)	0.200
Live birth rate	30.36 (34/112)	21.82 (36/165)	0.109	26.42 (14/53)	20.00 (14/70)	0.401
Early abortion rate	27.45 (14/51)	28.30 (15/53)	0.923	40.00 (10/25)	44.00 (11/25)	0.774
Abortion rate	31.37 (16/51)	32.08 (17/53)	0.939	44.00 (11/25)	44.00 (11/25)	1.000
Ectopic pregnancy rate	1.96 (1/51)	0 (0/53)	0.490	0 (0/25)	0 (0/25)	/

*P* < 0.05 was considered statistically significant

Comparisons of neonatal outcomes between the two groups stratified by 35 years of age are summarized in Table 4. When patients were in the same age category, there was no significant difference in terms of gestational age, birth weight, birth height, and rates of preterm birth, low birth weight, and very low birth weight between groups A and B.

**Table 4:** The neonatal outcomes of patients among groups A-B stratified by 35 years of age

	< 35 years of age			≥ 35 years of age		
	Group A	Group B	*P*	Group A	Group B	*P*
Cycles (n)	198	207		48	50	
Preterm birth (< 37 weeks)	8.08 (16/198)	11.59 (24/207)	0.236	10.42 (5/48)	12.00 (6/50)	0.804
Singleton	8.08 (16/198)	11.11 (23/207)	0.301	10.42 (5/48)	10.00 (5/50)	1.000
Twin	0 (0/198)	0.48 (1/207)	1.000	0 (0/48)	2.00 (1/50)	1.000
Gestational age (weeks)	38.56 ± 1.61	38.38 ± 1.76	0.421	38.27 ± 1.32	37.80 ± 2.37	0.209
Singleton	38.57 ± 1.61	38.41 ± 1.76	0.468	38.27 ± 1.32	38.20 ± 1.29	0.828
Twin	/	/		/	/	
Birth height (mm)	49.86 ± 1.90	49.82 ± 1.97	0.495	49.64 ± 1.70	49.48 ± 2.66	0.205
Singleton	49.90 ± 1.86	49.86 ± 1.95	0.423	49.64 ± 1.70	49.89 ± 1.79	0.856
Twin	/	/		/	/	
Birth weight (kg)	3224.27 ± 467.13	3186.73 ± 530.87	0.273	3303.65 ± 590.94	3221.96 ± 595.62	0.799
Singleton	3228.12 ± 467.88	3196.76 ± 530.90	0.316	3303.65 ± 590.94	3303.47 ± 443.37	0.179
Twin	/	/		/	/	
Low birth weight (< 2500 g)	6.03 (12/199)	8.13 (17/209)	0.408	6.25 (3/48)	7.84 (4/51)	1.000
Singleton	6.03 (12/199)	8.13 (17/209)	0.408	6.25 (3/48)	3.92 (2/51)	0.945
Twin	0 (0/199)	0 (0/209)	/	0 (0/48)	3.92 (2/51)	0.495
Very low birth weight (< 1500 g)	0 (0/199)	0.48 (1/209)	1.000	0 (0/48)	3.92 (2/51)	0.495
Singleton	0 (0/199)	0.48 (1/209)	1.000	0 (0/48)	0 (0/51)	/
Twin	0 (0/199)	0 (0/209)	/	0 (0/48)	3.92 (2/51)	0.495

*P* < 0.05 was considered statistically significant

## Discussion

This retrospective study is the first to compare the pregnancy outcomes between poor-quality D5 blastocysts and high-quality D6 blastocysts and to explore the transfer strategies of D6 high-quality blastocysts stratified by 35 years. We hope to provide more appropriate strategies for existing transfer strategies. Our results showed that the live birth rate of single poor-quality D5 blastocysts was higher than that of single high-quality D6 blastocysts, but the difference did not reach statistical significance for patients aged < 35 years (35.48% vs. 31.13%), and the same trend was observed for ≥ 35-year-old patients (29.09% vs. 21.28%). Additionally, single good-quality D6 blastocyst transfer can be considered for the acceptable pregnancy outcomes.

SBT is an effective method for reducing the risk of multiple pregnancies. To evaluate whether patients are suitable for SBT, many factors that affect the pregnancy outcomes of blastocyst transfer cycles must be comprehensively considered, including age, blastocyst developmental speed, and morphological grading. Numerous studies have investigated the effect of blastocyst developmental speed on pregnancy outcomes and showed that the rates of ongoing pregnancy and live birth of D5 blastocysts were significantly higher than those of D6 blastocysts [18, 19], which suggested that the timing of blastulation was considered as a stable predictor of live birth for frozen-thawed SBT [6]. A meta-analysis also showed that D5 blastocysts were superior to D6 blastocysts in both fresh and frozen transfer cycles in clinical practice [10, 11]. When transferring the same quality blastocysts, the live birth rate of D5 blastocysts in FET cycles was still significantly higher than that of D6 blastocysts [9, 20]. Additionally, Yerushalmi et al. found that D5 blastocysts in the oocyte donation program resulted in significantly better clinical outcomes and shorter time to delivery than D6 blastocysts, regardless of embryo quality [21]. One potential mechanism is that blastocyst developmental speed is associated with the incidence of abnormal spindle morphology. Hashimoto et al. showed that the incidence of abnormal spindles in the D6 blastocysts was significantly higher than that in D5 blastocysts (47% vs. 30%, *p* < 0.05), and the implantation competence was decreased in D6 blastocysts compared with D5 blastocysts when a total of 533 spindles were analyzed on days 5 and 6 vitrified-warmed blastocysts [22].

Another possible reason is that earlier blastocyst development is independently associated with a higher likelihood of embryonic euploidy, which supports the preferential selection of normally developing D5 blastocysts for transfer to improve pregnancy outcomes compared to growth-retarded D6 blastocysts among unbiopsied embryos [23, 24]. However, Kaye et al. found that D6 blastocysts resulted in similar clinical and ongoing pregnancy rates compared to those of D5 blastocysts after vitrification [25]. Yang et al. showed that for unbiopsied embryos, the clinical pregnancy rate of high-quality D5 blastocysts did not differ significantly from that of high-quality D6 blastocysts [26]. Similarly, for biopsied embryos, D5 euploid blastocysts showed no significant difference in implantation potential and early miscarriage rate compared with similarly graded day 6 euploid blastocysts [12]. Nevertheless, a study by Irani et al. showed that D5 blastocysts yielded a significantly higher live birth rate than similarly graded euploid D6 blastocysts [27]. Therefore, there is no consensus on the effect of blastocyst developmental speed on pregnancy outcomes, which may be caused by variant populations included and different quality of blastocysts transferred, as well as different blastocyst culture strategies and methods in different centers.

In addition to aneuploidy testing and blastocyst development rate, blastocyst morphological grading is critical for selecting optimal embryos with a good development potential for implantation [27]. For unbiopsied blastocyst transfer cycles, high-quality blastocysts were associated with a significantly higher live birth rate than low-quality blastocysts [20]. Our previous study is consistent with this conclusion and showed that the live birth rate of good-quality D5-SBT was significantly higher than that of low-quality D5-SBT [15]. These conclusions may be explained by the higher euploidy rates of high-quality blastocysts than low-quality blastocysts [28]. In cases of euploid blastocyst transfer, the live birth rates of high-quality, average-quality, and poor-quality blastocysts were 67.8%, 53.4%, and 29.5%, respectively, with a statistically significant difference [27]. Additionally, the clinical pregnancy rate of high-quality blastocysts was significantly higher than that of poor-quality blastocysts (54.5% vs. 25.0%, *p* = 0.034), and similar trend also exists with blastocysts at the same developmental speed [12]. Therefore, these studies suggest that, whether embryo biopsy is performed or not, blastocysts with high morphological grading should be preferentially selected for transfer to obtain better pregnancy outcomes. However, how can SBT be performed for better pregnancy outcomes when faced with D5 poor-quality and D6 high-quality blastocysts? Our results showed that the live birth rate of D5 poor-quality blastocysts was higher than that of D6 high-quality blastocysts for patients aged < 35 years (35.48% vs. 31.13%), and the same trend was observed for patients aged ≥ 35 years (29.09% vs. 21.28%), suggesting that D5 poor-quality blastocysts may be preferred, especially for advanced age patients, although there was no statistical difference.

To date, only a limited number of studies have explored transfer strategies for high-quality D6 blastocysts. A study by Kang et al. showed that D6-SBT produced significantly lower clinical outcomes compared with D6-DBT (23.4% vs. 51.6%, *p* = 0.001) when patients were transferred with high-quality blastocysts in FET cycles [29]. However, this study did not analyze results stratified by age, a factor that has a significant impact on pregnancy outcomes. Kaing et al. showed that older maternal age (*p* < 0.0001) is independently associated with a lower euploidy percentage [23]. Another study suggested that the quality of blastocysts degraded and the proportion of high-quality blastocysts decreased as age increased [14]. When blastocysts developed into the same quality, clinical pregnancy and live birth rates tended to decrease with increasing age [14]. Therefore, multiple embryo transfers are considered to improve pregnancy outcomes in elderly patients. Our previous study showed that SBT of D5 blastocysts could be recommended for advanced women, owing to significantly reduced multiple pregnancy rate and acceptable live birth rate compared to DBT, regardless of blastocyst quality [15]. For poor-quality D6 blastocysts, DBT is recommended for transfer to improve the live birth rate [16]. However, it is unclear whether SBT is adequately recommended for high-quality D6 blastocysts, especially in elderly patients. This study showed that for patients aged < 35 years, SBT of good-quality D6 blastocysts can be suggested for a live birth rate of 31.13%. And for patients ≥ 35 years of age, SBT of high-quality D6 blastocysts also could be recommended for an acceptable live birth rate of 21.28%.

There is no consensus regarding the effects of blastocyst developmental speed and morphological grading on neonatal outcomes. Previous studies have shown that D6 blastocyst transfer is associated with increased birth weight after FET compared with that in the D5 group [9, 30], but there was no difference in gestational age between D5 and D6 blastocysts [30]. Meanwhile, the conclusion of the meta-analysis was consistent with the above-mentioned study [31]. However, Wang et al. showed that birth weight in singleton newborns of the D5 blastocyst group exhibited no significant difference from that of the D6 group [32]. When blastocyst quality was considered, single poor-quality blastocyst transfer had significantly lower birth weight and gestation-adjusted birth weight than SBT of high-quality blastocysts during FET cycles [33]. However, Oron et al. found that embryo quality was not associated with adverse obstetric and perinatal outcomes, with no significant differences in gestational age and birth weight between single good- or poor-quality embryo [34]. Similar to the above-mentioned study, our study showed that the gestational age and birth weight of D5 poor-quality blastocyst transfer were not significantly different from those of D6 high-quality blastocyst, and this non-statistically significant result may be possibly due to the limited population included in this study. Larger-scale population studies are needed to confirm the effects of blastocyst developmental speed and quality on neonatal outcomes.

This study had some limitations. First, it was a retrospective study design; however, the baseline characteristics of the patients among the two groups were not statistically different, indicating that the populations included in this study among these groups were similar. Second, there was a relatively inadequate sample size of live births, resulting from patients aged ≥ 35 years, and the results from women of advanced age need to be verified in further large population studies. Third, patients over 40 years of age were not recruited in this study, which may prevent generalization of the conclusions. The main strength of this study was that the FET cycles were specifically grouped based on a combination of morphological grading and blastocyst developmental speed, and data were analyzed among groups stratified by 35 years of age, which may help improve existing blastocyst selection strategies.

## Conclusion

Our results suggest that poor-quality D5 blastocyst transfer was preferentially selected over high-quality D6 blastocysts, especially for advanced patients. SBT of D6 good-quality blastocysts was recommended for the acceptable pregnancy outcomes.

## Data Availability

The raw data supporting the conclusions of this article are not publicly available because they involve patient privacy, but are available from the corresponding author upon reasonable request.

## Abbreviations

DBT: Double blastocyst transfe
FET: Frozen embryo transfe
GnRH: Gonadotropin-releasing hormone
IVF-ET: In vitro fertilization and embryo transfer
SBT: Single blastocyst transfe
